# Inter-Genera Colonization of *Ocimum tenuiflorum* Endophytes in Tomato and Their Complementary Effects on Na^+^/K^+^ Balance, Oxidative Stress Regulation, and Root Architecture Under Elevated Soil Salinity

**DOI:** 10.3389/fmicb.2021.744733

**Published:** 2021-10-18

**Authors:** Pramod K. Sahu, Shailendra Singh, Udai B. Singh, Hillol Chakdar, Pawan K. Sharma, Birinchi K. Sarma, Basavaraj Teli, Raina Bajpai, Arpan Bhowmik, Harsh V. Singh, Anil K. Saxena

**Affiliations:** ^1^ICAR-National Bureau of Agriculturally Important Microorganisms, Maunath Bhanjan, India; ^2^Department of Mycology and Plant Pathology, Institute of Agricultural Science, Banaras Hindu University, Varanasi, India; ^3^ICAR-Indian Agricultural Statistics Research Institute, New Delhi, India

**Keywords:** endophytes, salinity, inter-genera colonization, root architecture, *Bacillus*, oxidative stress, reactive oxygen species, Na^+^/K^+^ balance

## Abstract

Endophytic bacilli of ethano-botanical plant *Ocimum tenuiflorum* were screened for salt stress-alleviating traits in tomato. Four promising *O. tenuiflorum* endophytes (*Bacillus safensis* BTL5, *Bacillus haynesii* GTR8, *Bacillus paralicheniformis* GTR11, and *Bacillus altitudinis* GTS16) were used in this study. Confocal scanning laser microscopic studies revealed the inter-genera colonization of *O. tenuiflorum* endophytes in tomato plants, giving insights for widening the applicability of potential endophytes to other crops. Furthermore, in a pot trial under 150 mM NaCl concentration, the inoculated endophytes contributed in reducing salt toxicity and improving recovery from salt-induced oxidative stress by different mechanisms. Reduction in reactive oxygen species (ROS) (sub-cellular H_2_O_2_ and superoxide) accumulation was observed besides lowering programmed cell death and increasing chlorophyll content. Endophyte inoculation supplemented the plant antioxidant enzyme system *via* the modulation of enzymatic antioxidants, *viz.*, peroxidase, ascorbate peroxidase, superoxide dismutase, and catalase, apart from increasing proline and total phenolics. Antioxidants like proline have dual roles of antioxidants and osmoregulation, which might also have contributed to improved water relation under elevated salinity. Root architecture, *viz*., root length, projection area, surface area, average diameter, tips, forks, crossings, and the number of links, was improved upon inoculation, indicating healthy root growth and enhanced nutrient flow and water homeostasis. Regulation of Na^+^/K^+^ balance and water homeostasis in the plants were also evident from the modulation in the expression of abiotic stress-responsive genes, *viz*., *LKT*1, *NHX*1, *SOS*1, *LePIP*2, *SlERF*16, and *SlWRKY*39. Shoot tissues staining with light-excitable Na^+^ indicator Sodium Green^TM^ Tetra (tetramethylammonium) salt showed low sodium transport and accumulation in endophyte-inoculated plants. All four endophytes exhibited different mechanisms for stress alleviation and indicated complementary effects on plant growth. Furthermore, this could be harnessed in the form of a consortium for salt stress alleviation. The present study established inter-genera colonization of *O. tenuiflorum* endophytes in tomato and revealed its potential in maintaining Na^+^/K^+^ balance, reducing ROS, and improving root architecture under elevated salinity.

## Introduction

Increasing soil salinity has become a major bottleneck in realizing the production potential of crops. A recent report indicated that 11.73 million km^2^ soil area is salt affected, in which Asia, Africa, and Australia are worst affected (Hassani et al., 2020). In saline soils, high osmotic stress and sodium toxicity adversely affect nutrient uptake, mobilization, osmotic balance, membrane integrity, oxidative stress, rate of photosynthesis, and overall growth, thereby seriously limiting crop productivity and limiting sustainable land use (Czarnocka and Karpiński, 2018; Arif et al., 2020; Pan et al., 2020). Breeding-based approaches for the alleviation of salt stress have several bottlenecks (Uga, 2021). The amelioration strategies for reducing soil salinity are cost intensive (Qadir et al., 2001), and the genetically modified crops have a long way to go to realize their widespread usage for salinity tolerance in major crops (Kumar et al., 2020). However, microbial agents, specifically the bacterial endophytes, were found to have salinity tolerance, thus improving capability (Abdelshafy Mohamad et al., 2020; Kushwaha et al., 2020).

Microbes have different mechanisms for abiotic stress management, such as maintaining Na^+^/K^+^ balance (Lee et al., 2016), spatial exclusion of ions *via* ion transporters (Zhang et al., 2008), restricting Na^+^ uptake in roots (Ashraf et al., 2004; Dodd and Pérez-Alfocea, 2012), osmotic balance (López-Gómez et al., 2019; Nadeem et al., 2020), membrane stability (Bano and Fatima, 2009; Singh and Jha, 2016; Yasin et al., 2018; Chauhan et al., 2019), improved nutrient uptake (Lee et al., 2015; Albdaiwi et al., 2019; Abdelshafy Mohamad et al., 2020), production of enzymatic (Halo et al., 2015; Fukami et al., 2018) and non-enzymatic antioxidants (Fazal and Bano, 2016; Singh and Jha, 2017), etc. Since all these effects are generally not found in a single endophyte, exploring the endophytes having different complementary mechanisms could pave the way for the inoculants having multiple mechanisms and stress-alleviating potential.

Endophytes exhibit enormous diversity even at the cultivar level (Sahu et al., 2020b); therefore, exploring different plants and their varieties for potential salt stress alleviation is desirable. Holy basil (*Ocimum tenuiflorum*), also called “Tulsi” locally, is a medicinal shrub having a religious value (Shekhawat and Shah, 2013; Singh and Chaudhuri, 2018; Taufiq and Darah, 2018). The biological control potential of bacterial endophytes from *O. tenuiflorum* has been previously worked out (Chowdhary and Kaushik, 2015; Sahu et al., 2020a) along with their potential to alleviate oxidative stress. However, there are only scanty reports available for the application of *O. tenuiflorum* endophytes in abiotic stress alleviation. Since there is a close cross-talk between signaling for biotic and abiotic stresses in plants (Fujita et al., 2006) and oxidative stress arises from both biotic and abiotic stresses, therefore, the present investigation was carried out for exploring *O. tenuiflorum* endophytes for alleviation of salt stress in tomato.

On the other hand, the horizontal transmission of the endophytes from the surrounding to the system of the plant is crucial for the success of applied endophytic inoculants (Verma et al., 2021b). Magnificent studies have been done on the potential of endophytic inoculants (Egamberdieva et al., 2016; Abdelshafy Mohamad et al., 2020); nevertheless, the adaptability on different crop hosts was least worked out. Endophytic microbes can confer salt tolerance ability to their host plants (Khan et al., 2020). Whether the same kind of protection could be conferred to the plants other than the host needs to be explored for wider adaptability and further commercialization of endophytic strains. The use of endophytes, which are able to successfully colonize the non-native host and improve the plant performance, could be of greater significance for the utility of commercial microbial inoculants on a wide range of crops. Keeping all these considerations, the present study was conducted to explore *O. tenuiflorum* bacterial endophytes for inter-genera colonization in tomato, their effect, and complementation on salt stress-mitigating strategies of plant.

## Materials and Methods

### Source of Bacterial Endophytes Used in the Study

*O. tenuiflorum* bacterial endophytes from our previous study (Sahu et al., 2020a), namely, *Bacillus safensis* BTL5, *Bacillus haynesii* GTR8, *Bacillus paralicheniformis* GTR11, and *Bacillus altitudinis* GTS16 (National Agriculturally Important Culture Collection, ICAR-NBAIM, India accession numbers NAIMCC-B-02221–NAIMCC-B-02224, respectively), were used for the *in vivo* studies. Two *in vivo* experiments were conducted: the first was to confirm inter-genera endophytic colonization in tomato plants in a seedling tray experiment and the second was to evaluate the salt stress alleviation potential of these endophytes in pot trial.

### Inter-Genera Colonization Study

#### Growing Plants in Seedling Trays

Tomato seeds (variety, Pusa Ruby) were surface sterilized by washing in sterile water, followed by dipping in 70% ethanol for 30 s and washing with sterile distilled water. Afterward, the seeds were dipped in 2% sodium hypochlorite (1 min) followed by three sterile distilled water washes, followed by 10-min dip in 2% sodium thiosulfate. The surface-sterilized seeds were treated with the respective bacterial endophyte inoculum (24 h old, 0.2 OD suspension). Six treatments were made, each with 10 replications. The treatment details were as follows: T1 = bbsolute control, T2 = saline control (150 mM NaCl), T3 = 150 mM NaCl + *Bacillus safensis* BTL5, T4 = 150 mM NaCl + *Bacillus haynesii* GTR8, T5 = 150 mM NaCl + *Bacillus paralicheniformis* GTR11, and T6 = 150 mM NaCl + *Bacillus altitudinis* GTS16. The seeds were sown in 98-well seedling trays containing sterile sand: soil mixture (1:3). Nutrients were provided through sterile Hoagland solution (1X). The tomato plants were allowed to grow for a period of 3 weeks before harvesting the roots for the colonization studies.

#### Visualization of Endophytic Colonization

The roots were gently washed thrice in sterile distilled water and stained with LIVE/DEAD^TM^ *Bac*Light^TM^ bacterial viability stain (Invitrogen, United States) containing SYTO9 and propidium iodide (Stiefel et al., 2015). The roots were stained with 35-μM concentration of SYTO9 and 400 μM of propidium iodide for a period of 30 min in the dark. The excess fluorescent dyes were washed using phosphate buffer saline. The stained roots were mounted on glass slides and visualized under a confocal scanning laser microscope (CSLM; Nikon, Japan) using 488 nm, 543 nm, and TD channels. The images were processed using NIS element 3.2.3 program (Nikon, Japan).

### *In vivo* Evaluation of Salinity Tolerance in Tomato

#### Soil Preparation and Nursery

Pots were filled with 5 kg of autoclaved soil. The heterogeneity within the pots was nullified by row and column randomization. The nutrients were mixed in a ratio of 100:50:50 kg NPK per hectare using urea, diammonium phosphate, and muriate of potash. The soil in these pots was brought to natural compaction by three cycles of wetting and drying. Salinity was maintained by 150 mM NaCl, and the pots were irrigated till 80% of the field capacity. Tomato (variety, Pusa Ruby) seeds were sown in a planting tray containing soil/sand/cocopit mixture (1:1:1) and kept moistened by sprinkling water. After 30 days, the plants were removed; the roots were gently washed and treated with the respective endophytic inoculant by root dipping. The inoculum (5 ml/L; 24-h-old 0.2 OD culture of respective endophytes) was mixed with 0.5% carboxy methyl cellulose, and the roots were dipped for half an hour and transplanted in the pots. The treatment details were the same as used in the seedling tray experiment with three replications. This experiment was repeated twice for the consistency of the results under a similar experimental setup. Data was recorded 45 days after transplanting (DAT); however, root architecture, length, and dry weight of the plant was measured 75 DAT.

#### Modulation in Gene Expression

##### RNA Extraction and cDNA Preparation

The plant sample (100 mg of root, shoot, and leaf) was mixed with 1 ml TRIzol reagent and 10 μl mercaptaethanol and incubated for 5 min at ambient temperature before grinding in liquid nitrogen and further processed as per the instructions of the manufacturer for using PureLink^TM^ RNA mini-Kit (Invitrogen, United States). The total RNA isolated was converted to cDNA using High Capacity RNA-to-cDNA kit (Thermo Fisher Scientific, United States) following instructions of the manufacturer. The cDNA was further used for real time quantitative PCR (RT-qPCR) studies.

##### RT-qPCR Assay

The RT-qPCR based expression analyses of salt stress-responsive genes (Table 1) were carried out using RT-qPCR Detection System (Bio-Rad, United States) and Eva Green SYBR Green Supermix Kit (Bio-Rad, United States) using three technical replicates of each sample. The final concentration of gene-specific primers was maintained at 10 pmol/μl, and internal controls were utilized to determine and normalize the transcript level of mRNA. The final volume of the reaction mixture was maintained to 10 μl [diluted cDNA samples (50 ng/ml), 2 μl; forward and reverse primers (10 μM), 1.5 μl each; and real-time master mix, 5 μl]. The RT-qPCR condition involved denaturation at 95°C for 2 min, 40 repeats at 95°C for 30 s, 60°C for 30 s, and 72°C for 30 s. The 2^–ΔΔCT^ method was used for data normalization using the mean CT values of the two endogenous genes, actin and glyceraldehyde 3-phosphate dehydrogenase. The fold accumulation of transcripts was compared by using the mean of the CT values of the three technical replicates from each biological replication with control (Livak and Schmittgen, 2001).

**TABLE 1:** List of RT-qPCR primers used in the gene expression analysis.

S. no.	Genes	Primer sets	Sequence (5′–3′)	References
1.	K^+^ transporter	*LKT*1-F	ACTTGCCTCTCACCCTTTG	Wei et al., 2017
		*LKT*1-R	CCACACAGTTCTCAATGCC	
2.	Aquaporin	*LePIP*2-F	AAGGATTACAAAGAGCCACC	Shiota et al., 2006
		*LePIP*2-R	ACCAAAAGCCCAAGCAAC	
3.	Na^+^/ K^+^ antiporter	*NHX*1-F	TGGCGAGATTGGGGGTGAGT	Wei et al., 2017
		*NHX*1-R	AACGACTCTCTTCAAGGAGATGACC	
4.	Ethylene response factor transcription factor	*SlERF* 16-F	GCGAATAATACAGAACCCGAACTT	Gharsallah et al., 2016
		*SlERF* 16-R	TGAGGAAGAAGAAAGATCCGAATT	
5.	WRKY transcription factor	*SlWRKY* 39-F	GCGGTAATGCCAAGACAAAC	Gharsallah et al., 2016
		*SlWRKY* 39-R	TCAGTTCCTGGTGATTTACGC	
6.	Salt overly sensitive Na^+^/K^+^ antiporter	*SOS*1-F	GTGCAGTACAGATGCTTTTACTTG	Wei et al., 2017
		*SOS*1-R	AGGGCCACAACAGCCACAG	
7.	Actin (reference gene)	*Actin*-F	GGAACTTGAGAAGGAGCCTAAG	Wei et al., 2017
		*Actin-*R	CAACACCAACAGCAACAGTCT	
8.	GAPDH (reference gene)	*gadph-*F	GAAATGCATCTTGCACTACCAACTGTCTTGC	Shih et al., 1992
		*gadph-*R	CTGTGAGTAACCCCATTCATTATCATACCAAGC	

#### Cellular Osmotic Balance and Membrane Integrity Under Elevated Salinity

##### Total Phenolics

The accumulation of total phenolic content (TPC) in the plants was assessed by the protocol described by Malick and Singh (1980) and Sadasivam and Manickam (1996). One gram of leaf sample was ground in pestle and mortar using 80% ethanol. The content was centrifuged at 10,000 rpm for 20 min. The supernatant was evaporated, and the precipitate was dissolved in 5 ml distilled water. Then, 200 μl of this content was made up to 3 ml with distilled water, and 0.5 ml of Folin–Ciocalteau reagent was added. Two milliliters of 20% sodium carbonate was added after 3 min and mixed thoroughly. It was kept in boiling water bath for 1 min, and OD was recorded at 650 nm.

##### Proline Content

Proline content was measured by crushing 0.5 g of leaf sample in 10 ml of 3% aqueous sulphosalicylic acid. In 2 ml of the filtrate, 2 ml each of glacial acetic acid ninhydrin was added and kept in boiling water bath for 1 h. After reaction termination, toluene (4 ml) was mixed and kept at ambient temperature for toluene layer separation. The upper layer was taken, and the absorbance of red color was measured at 520 nm. Calculation was done using a standard curve as described by Sadasivam and Manickam (1996).

##### Electrolyte Leakage

Ten leaf discs were placed in 25 ml of distilled water and kept for 4 h at ambient temperature to measure the electrical conductivity. The same was autoclaved at 121°C for 30 min, and the electrical conductivity was measured. The electrolyte leakage was assessed following the formula given in Khare et al. (2010).

#### Effects of *O. tenuiflorum* Endophyte Inoculation on Enzymatic Reactive Oxygen Species Scavengers

The activity of peroxidase (PO), catalase (CAT), superoxide dismutase (SOD), and ascorbate peroxidase (APx) was measured at 50 DAT as per the protocol given in Sadasivam and Manickam (1996). Briefly, enzyme extract (for all four enzymes) was prepared by grinding 1 g of fresh plant tissue in phosphate buffer (0.1 M, 3 ml, pH 7.0) and centrifuged at 12,000 rpm for 15 min. For PO activity, the 100-μl enzyme extract was mixed with 50 μl of 20 mM guaiacol solution, and 3 ml of 50 mM phosphate buffer was followed by the addition of 30 μl of 12.3 mM H_2_O_2_ in a cuvette to start the reaction; the absorbance was recorded at 436 nm. For CAT activity, the reaction mixture was prepared by mixing 100 μl of enzyme extract and 3 ml of 50 mM phosphate buffer, followed by the addition of 30 μl of 12.3 mM H_2_O_2_ in a cuvette to start the reaction; the absorbance was recorded at 240 nm. For SOD activity, 50 μl of enzyme extract was added to the reaction mixture containing 50 mM phosphate buffer, pH 7–8, 13 mM methionine, 75 mM nitroblue tetrazolium (NBT), 2 mM riboflavin, and 1 mM EDTA. In this mixture, riboflavin was added last, and the tubes were shaken. The reaction was started by placing these tubes under light for 10 min and stopped by covering the tubes with a dark cloth, and the absorbance was read at 560 nm. For APx activity, 1 ml of reaction mixture containing 50 mM KPO_4_ buffer (pH 7.0), crude enzyme extract, and 0.35 mM ascorbate were prepared. The reaction was initiated by adding 5 μl of 10 mM H_2_O_2_. In this reaction mixture, the H_2_O_2_-dependent oxidation of ascorbate was recorded at 290 nm for 120 s.

#### Accumulation of Reactive Oxygen Species and Programmed Cell Death

##### H_2_O_2_ Accumulation

Detection of H_2_O_2_ accumulation was done by staining with 3,3-diaminobenzidine (DAB; Sigma) following the protocol of Sakamoto et al. (2008). Leaf tissue was dipped in DAB solution overnight, and polymerization of DAB could be seen as a brown color deposit after decoloration with ethanol/glacial acetic acid (3:1). The images were captured using a stereomicroscope.

##### Superoxide Accumulation

The superoxide radical (O_2_^–^) present in the leaf tissue reacts with nitroblue tetrazolium (NBT; Sigma) and forms blue-colored formazan (Rao and Davis et al., 1999). The NBT solution was prepared by dissolving 0.2 g NBT in 50 mM of sodium phosphate buffer (pH 5.5). The leaf tissues were dipped in this solution and incubated overnight, followed by bleaching with ethanol/glacial acetic acid (3:1) solution. The blue-colored spots, indicating superoxide radicals, appeared, and images were captured using a stereomicroscope.

##### Programmed Cell Death

Programmed cell death (PCD) in leaf tissue due to salt stress was assessed by Evans Blue staining (Baker and Mock, 1994). The leaf tissue was decolorized by boiling in ethanol, followed by dipping for 5–6 h in 0.25% of aqueous solution of Evans Blue for 5–6 h. The development of blue-colored spots indicated PCD. The images were captured using a stereomicroscope.

#### Na^+^ Transport in Plant Tissues

The transport of Na^+^ ions in shoot was assessed using Sodium Green^TM^ Tetra (tetramethylammonium) salt (Invitrogen, United States) by capturing the sodium fluorescence using a confocal scanning laser microscope. Shoot tissues of equal physiological states were taken, and thin sections were prepared using a microtome and stained with Sodium Green^TM^ Tetra (tetramethylammonium) salt. The stained sections were mounted in slides and visualized under a CSLM (Nikon 90i, Japan) using 488-nm channel. The images were processed using NIS element 3.2.3 program (Nikon, Japan). All the treatments were visualized using the same optical adjustment to avoid any capture artifacts in signal strength.

#### Chlorophyll Content

After 45 days of transplantation, leaves were collected for assessing chlorophyll content as described by Witham et al. (1971). One gram of leaf tissue was crushed in 80% pre-chilled acetone, and the volume was made up to 100 ml with pre-chilled acetone. The absorbance of the supernatant was recorded at 663 and 645 nm using UV–*vis* 1700 spectrophotometer (Shimadzu, Japan). The amount of chlorophyll present (mg/g) in the leaf tissue was calculated using the following formula:

(1) Chl *a* mg/g of leaf = 12.7 (*A*_663_) – 2.69 (*A*_645_) × V/(1,000 × w)
(2) Chl *b* mg/g of leaf = 22.9 (*A*_645_) – 4.68 (*A*_663_) × V/(1,000 × w)

#### Root Architecture

The entire soil from the pot was washed carefully to harvest the complete root system. The harvested roots were washed thrice with clean water to remove the adhering soil. The roots were placed on the scanning tray filled with sterile water. The roots were scanned using EPSON Expression 12000 XL scanner, and the subsequent analyses of various root parameters [like cumulative length of total root (cm), projection area (cm^2^), surface area (cm^2^), average diameter (mm), root volume (cm^3^), tips, forks, crossings, and number of links] were carried out using WINRHIZO Pro software.

### Statistical Analysis

In this study, laboratory experiments were laid out in a completely randomized design with three replications each and repeated thrice. The experiment for inter-genera colonization was carried out with six treatments and 10 replications. The pot experiment was laid out in a randomized complete block design with six treatments and three replications each, and the pot experiment was repeated twice. All the data (except for the gene expression study) were analyzed using statistical formulae in Microsoft Office Excel, and means were compared with Duncan’s multiple range test at *p* ≤ 0.05. Origin program was used for the graphical representation of data. The gene expression data was analyzed and compared based on non-parametric test (Kruskal–Wallis test) using SPSS 20 program.

## Results

### Inter-Genera Colonization of *O. tenuiflorum* Endophytes in Tomato Roots

**FIGURE 1:**
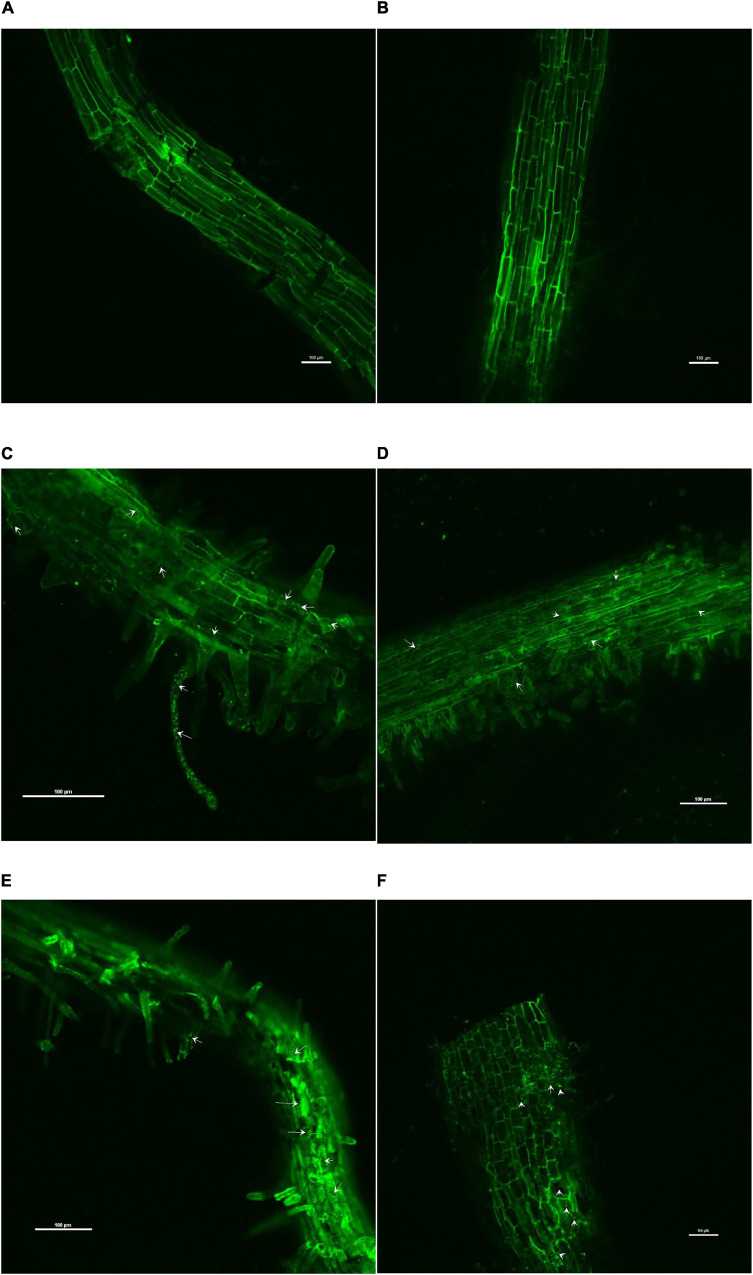
Confocal scanning laser microscopy images showing the inter-genera colonization of *Ocimum tenuiflorum* bacterial endophytes in tomato roots treated with LIVE/DEAD^TM^ *Bac*Light^TM^ bacterial viability stain. The white arrows indicate live bacterial endophytes in tomato roots in different treatments. **(A)** absolute control, **(B)** saline control, **(C)** BTL5, **(D)** GTR8, **(E)** GTR11, and **(F)** GTS16.

The confocal scanning laser micrographs of the colonization experiment showed that all the four endophytes could establish themselves in the tomato roots with variable colonization patterns (Figure 1). SYTO9 was present in LIVE/DEAD^TM^*Bac*Light^TM^ bacterial viability staining kit-stained bacterial cells in yellow-green color based on membrane potential and integrity. Bacterial signals were observed from the epidermis and root hair cells. *O. tenuiflorum* endophytes *Bacillus haynesii* GTR8 and *Bacillus altitudinis* GTS16 were found to colonize in the rhizoplane as well as endosphere, whereas *Bacillus paralicheniformis* GTR11 and *Bacillus safensis* BTL5 showed only internal colonization (Figures 1C–E). However, absolute and saline control plants had signals, too, but the intensity was relatively very less (Figures 1A,B).

### Modulation in Gene Expression

The treatments were significantly different for all the genes at 1% level of significance except in the case of *NHX*1, where treatments are significantly different at 5% level of significance. The *LKT*1 gene was over-expressed in *Bacillus altitudinis* GTS16-treated plants (17.17-folds), whereas statistically non-significant differences were found among other treatments (Table 2). The *NHX*1 gene expression was more than fourfolds higher in *Bacillus altitudinis* GTS16 inoculation and more than twofolds higher in *Bacillus safensis* BTL5 and *Bacillus haynesii* GTR8 inoculation, which was significantly higher than both absolute control and saline control. Overexpression of *SOS*1 by more than ninefolds was recorded in *Bacillus haynesii* GTR8 and *Bacillus altitudinis* GTS16 plants. However, there was no significant increase in other treatments. Although overexpression of *LePIP*2 gene was observed in all the saline treatments, the highest expression was recorded in *Bacillus paralicheniformis* GTR11. In *SlERF*16 and *SlWRKY*39 genes, a higher expression was recorded from *Bacillus haynesii* GTR8-inoculated treatments. *Bacillus altitudinis* GTS16 and *Bacillus paralicheniformis* GTR11 inoculation caused 3.49- and 2.17-folds increased in *SlERF*16 gene expression, respectively.

**TABLE 2:** Effect of bacterial endophytes on the expression of salt stress-responsive genes under elevated salinity (150 mM NaCl).

Treatments	*LKT*1		*LePIP*2		*NHX*1		*SOS*1		*SlERF*16		*SlWRKY*39	
	Fold change	Mean rank	Fold change	Mean rank	Fold change	Mean rank	Fold change	Mean rank	Fold change	Mean rank	Fold change	Mean rank
Absolute control	1.00^ab^	2	1.00^a^	1	1.00^ab^	2	1.00^ab^	2	1.00^b^	3	1.00^ab^	2
Saline control	1.62^b^	3	2.95^ab^	2	0.99^ab^	3	1.31^ab^	3	0.33^ab^	2	2.69^b^	3
BTL5	0.17^a^	1	3.57^ab^	3	2.14^bc^	5	0.18^a^	1	0.23^a^	1	0.43^a^	1
GTR8	3.24b^c^	5	35.40^c^	5	2.02^b^	4	9.93^d^	6	9.59^d^	6	31.13^d^	6
GTR11	1.71^c^	4	37.99^cd^	6	0.94^a^	1	2.18^b^	4	2.17^bc^	4	9.11^c^	5
GTS16	17.16^d^	6	8.29^b^	4	4.85^d^	6	9.71^c^	5	3.49^cd^	5	8.82^bc^	4

*The fold change data was analyzed and compared based on non-parametric test (Kruskal–Wallis test). Treatments with same superscript letters are at par. Treatments with letter “a” have the lowest expression. Ranks are given from lowest to highest (lowest expression ranked “1” and highest expression rated “6”).*

### Reactive Oxygen Species Accumulation in Tomato Leaves and Antioxidant System

#### Cellular Osmotic Balance and Membrane Integrity Under Elevated Salinity

**FIGURE 2:**
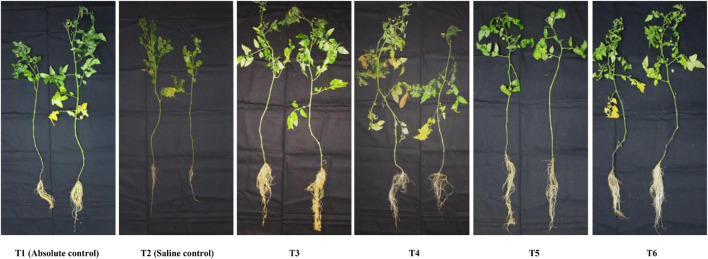
Phenotypic observation of the plants under different treatments indicating improvement in root and shoot growth and biomass accumulation. T1, absolute control; T2, saline control; T3, BTL5; T4, GTR8; T5, GTR11; and T6, GTS16.

**FIGURE 3:**
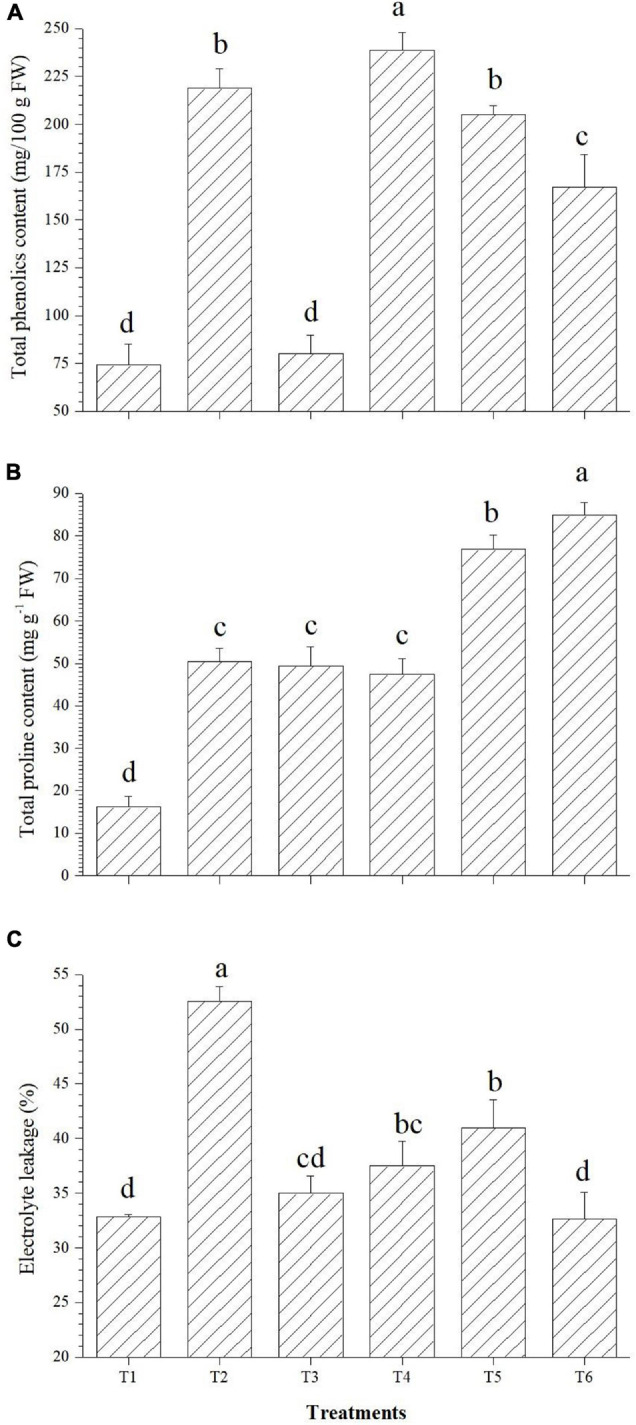
Effect of bacterial endophytes on **(A)** total phenolics content, **(B)** proline content, and **(C)** electrolyte leakage. T1, absolute control; T2, saline control; T3, BTL5; T4, GTR8; T5, GTR11; and T6, GTS16.

The applied bacterial endophytes were able to supplement plant mechinery for oxidative stress mitigation which were visible in the overall plant growth (Figure 2). The accumulation pattern of TPC was variable among the treatments. Treatment with *Bacillus haynesii* GTR8 inoculation accumulated a significantly higher TPC. On the contrary, treatments inoculated with *Bacillus safensis* BTL5 could accumulate lower levels of TPC at par with the absolute control plants (Figure 3A). In case of total proline, endophyte inoculation gave a mixed effect (Figure 3B). The *Bacillus altitudinis* GTS16- and *Bacillus paralicheniformis* GTR11-treated plants were having a higher accumulation, whereas *Bacillus safensis* BTL5 and *Bacillus haynesii* GTR8 were having a similar accumulation as saline control. However, both salinity and inoculation had significant effects on proline content. On the other hand, high salinity (saline control treatment) lowered the membrane integrity and enhanced the electrolyte leakage from tomato leaves (Figure 3C). Inoculation of endophytes *Bacillus altitudinis* GTS16 and *Bacillus safensis* BTL5 could improve the membrane integrity at par with the absolute control and significantly reduced the electrolyte leakage.

#### Effects of *O. tenuiflorum* Endophyte Inoculation on Enzymatic Reactive Oxygen Species Scavengers

**FIGURE 4:**
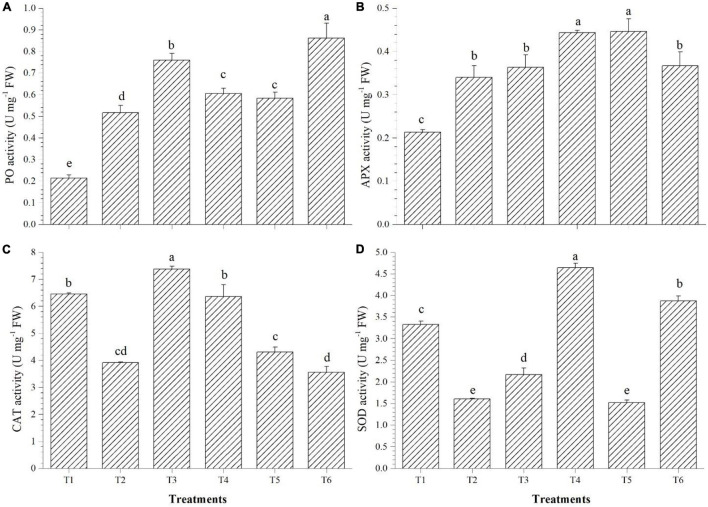
Effect of bacterial endophytes on enzymatic antioxidants under elevated salinity (150 mM NaCl). **(A)** Peroxidase, **(B)** ascorbate peroxidase, **(C)** catalase, and **(D)** superoxide dismutase. T1, absolute control; T2, saline control; T3, BTL5; T4, GTR8; T5, GTR11; and T6, GTS16.

PO accumulation was highest in T6 (3.45 U mg^–1^ FW), APx was higher in T4 (0.44 U mg^–1^ FW), CAT accumulated in highest quantity in T3 (7.38 U mg^–1^ FW), whereas SOD activity was found to be highest in the *Bacillus haynesii* GTR8-inoculated (4.64 U mg^–1^ FW) plants (Figures 4A–D). Inoculation of endophyte *Bacillus altitudinis* GTS16 remarkably enhanced the production of PO and SOD. *Bacillus haynesii* GTR8 inoculation was able to significantly enhance the levels of APx, CAT, and SOD than the positive control. Endophyte *Bacillus safensis* BTL5 could activate more of CAT and PO for alleviating the effects of salinity. *Bacillus paralicheniformis* GTR11 inoculation chiefly enhanced the levels of APx in tomato plants for reducing the effects of elevated salinity.

#### Accumulation of Reactive Oxygen Species and Programmed Cell Death

**FIGURE 5:**
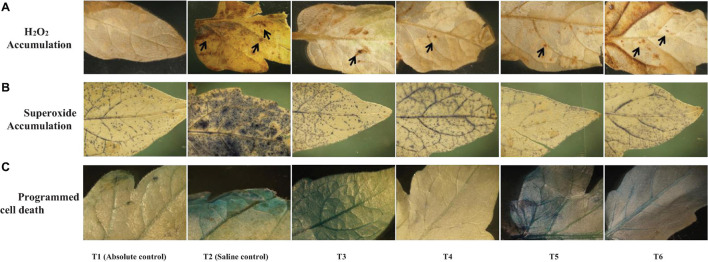
Effect of bacterial endophytes on **(A)** salinity induced sub-cellular H_2_O_2_ accumulation by 3,3′-diaminobenzidine, **(B)** superoxide accumulation by NBT staining, and **(C)** programmed cell death by Evan’s blue staining under elevated salinity (150 mM NaCl) as revealed. T1, absolute control; T2, saline control; T3, BTL5; T4, GTR8; T5, GTR11; and T6, GTS16.

The salinity induced sub-cellular H_2_O_2_ accumulation was reduced in the leaves of endophyte-inoculated plants, especially in *Bacillus safensis* BTL5-, *Bacillus haynesii* GTR8-, and *Bacillus paralicheniformis* GTR11-inoculated plants (Figure 5A). Superoxide accumulation was highest in the saline control. However, the accumulation was also observed in other treatments, including endophyte-inoculated plants, but was lesser than the absolute control treatment (Figure 5B). Evans Blue staining indicated increased PCD in the saline control and *Bacillus safensis* BTL5-, *Bacillus paralicheniformis* GTR11-, and *Bacillus altitudinis* GTS16-inoculated plants. Least PCD was observed from absolute control and *Bacillus haynesii* GTR8-inoculated plants (Figure 5C).

### Na^+^ Transport and Accumulation in Plant Tissues

**FIGURE 6:**
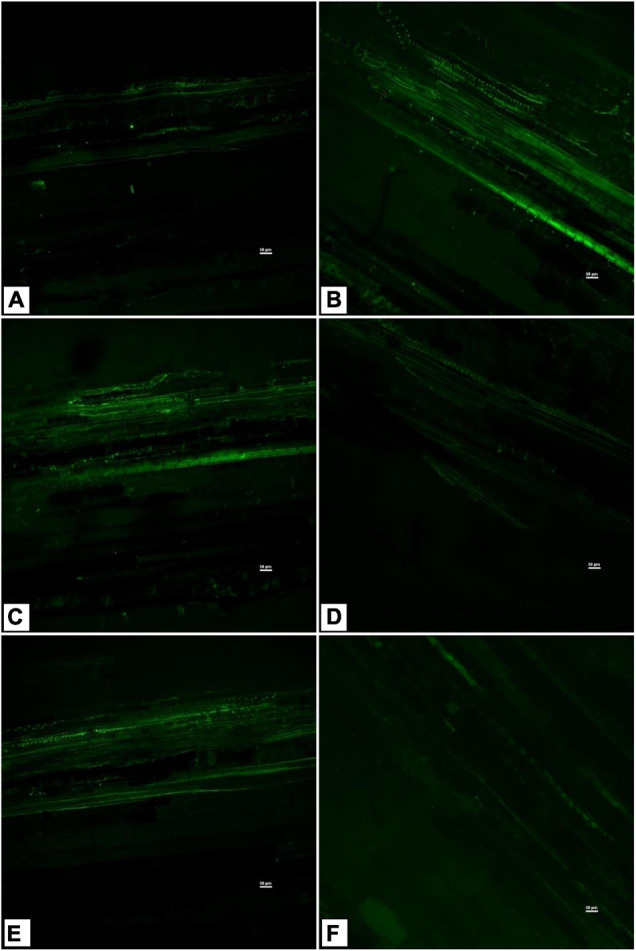
Confocal scanning laser microscopy images showing radial Na^+^ transport in shoot tissues stained with light-excitable Na^+^ indicator Sodium Green^TM^ Tetra (Tetramethylammonium) Salt. **(A)** Absolute control, **(B)** saline control, **(C)** BTL5, **(D)** GTR8, **(E)** GTR11, and **(F)** GTS16.

Sodium Green^TM^ staining of tomato shoot tissues indicated higher sodium transport and accumulation in the saline control treatment (Figure 6B) compared to the absolute control (Figure 6A). However, there were variations in endophyte-inoculated saline treatments (Figures 6C–F). Na^+^ transport and accumulation were less in *Bacillus haynesii* GTR8, *Bacillus paralicheniformis* GTR11, and *Bacillus altitudinis* GTS16, whereas *Bacillus safensis* BTL5 had higher sodium accumulation than other endophyte-inoculated treatments. *Bacillus haynesii* GTR8 plants had lowest Na^+^ transport, which was comparable to the absolute control (Figure 6D).

### Effect on Chlorophyll Content

**FIGURE 7:**
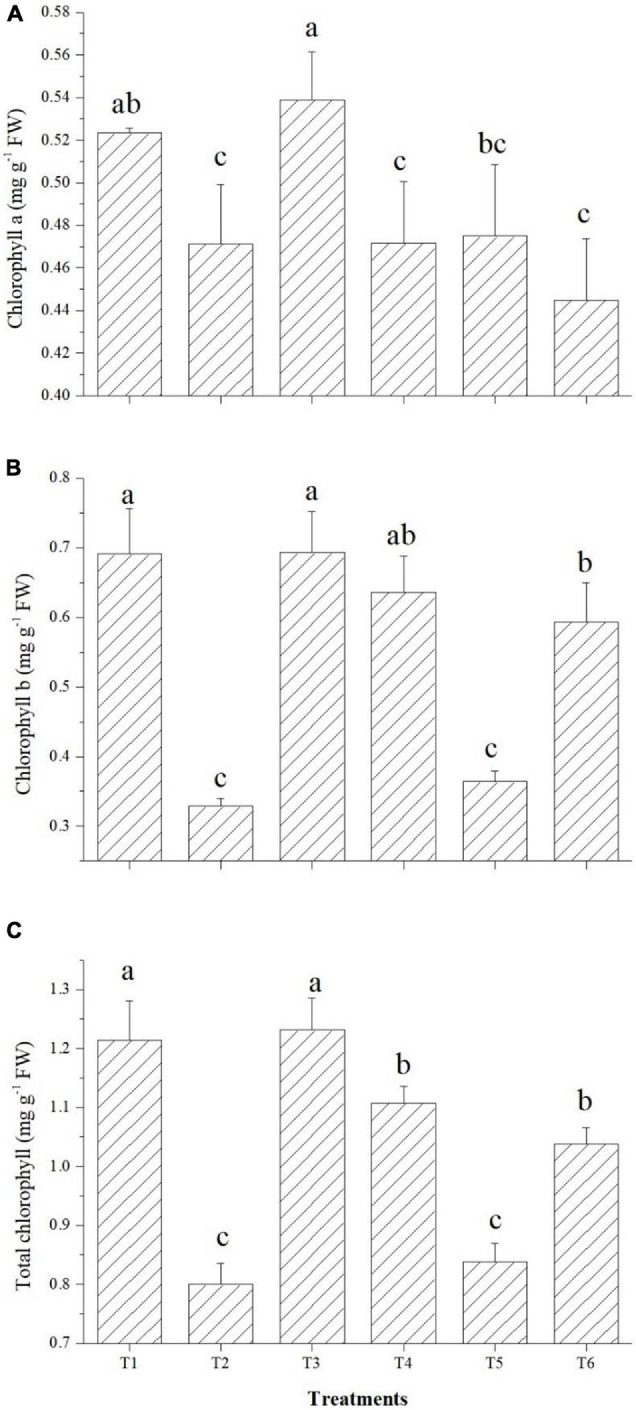
Effect of bacterial endophytes on chlorophyll content under elevated salinity (150 mM NaCl). **(A)** Chlorophyll *a*, **(B)** chlorophyll *b*, and **(C)** total chlorophyll. T1, absolute control; T2, saline control; T3, BTL5; T4, GTR8; T5, GTR11; and T6, GTS16.

The effects on the chlorophyll content of tomato leaves are shown in Figure 7. Among the endophytes, except *Bacillus paralicheniformis* GTR11, all three could improve the total chlorophyll content over the saline control. Endophyte *Bacillus safensis* BTL5 inoculation increased the total chlorophyll content of the tomato plants at par with the absolute control (Figure 7). In case of Chl *a* and Chl *b* also, significantly higher total chlorophyll content was observed in *Bacillus safensis* BTL5 inoculated and absolute control plants, whereas lowest was recorded from saline control plants.

### *O. tenuiflorum* Endophytes Modulated Tomato Root Architecture Under Elevated Salinity

Root scanning data for cumulative length of root, projection area, surface area, average root dia, root volume, number of tips, forks, crossings, and links were taken for comparison. The inoculation of endophytes resulted in a robust root system in terms of the cumulative length of the root, projection area, surface area, root volume, number of tips, forks, links, and crossings. However, no significant difference in root diameter was observed among various microbial inoculations and uninoculated controls. The inoculation of *Bacillus paralicheniformis* GTR11 resulted in the highest cumulative length of root (1,378.22 cm), projection area (67.87 cm^2^), surface area (213.23 cm^2^), tips (3,208.67), and crossings (1,887.0) among the microbial inoculation treatments (Table 3). It is worthy to note that the root system developed in the endophyte-inoculated tomato plants under salt stress was statistically comparable to un-inoculated plants grown without any stress.

**TABLE 3:** Effect of endophyte inoculation and salt stress on root architecture.

Treatments	Cumulative length of root^a^ (cm)	Projection area (cm^2^)	Surface area (cm^2^)	Average diameter (mm)	Root volume (cm^3^)	Tips	Forks	Crossings	Number of links
Absolute control	1,343.54 ± 101.30a	74.16 ± 4.36a	232.97 ± 13.69a	0.56 ± 0.07a	3.58 ± 0.22a	3,473.33 ± 430.60a	6,991.67 ± 701.58a	1,874.00 ± 176.69a	13,519.00 ± 1,201.05a
Saline control	538.10 ± 93.23c	22.32 ± 3.67c	70.13 ± 11.54c	0.42 ± 0.01b	0.73 ± 0.11d	1,865.50 ± 541.50c	1,954.00 ± 142.00c	571.50 ± 19.50c	4,249.00 ± 486.00c
BTL 5	1,111.01 ± 224.61ab	63.20 ± 28.32ab	198.56 ± 88.97ab	0.55 ± 0.16ab	4.05 ± 0.31a	2,582.67 ± 612.98abc	5,258.67 ± 1,774.71ab	1,499.67 ± 440.67ab	10,193.67 ± 2,956.31ab
GTR8	973.57 ± 189.01b	44.48 ± 11.24bc	139.75 ± 35.32bc	0.45 ± 0.04ab	1.94 ± 0.47c	2,776.33 ± 1,046.97ab	4,135.00 ± 1,345.62b	1,182.67 ± 382.62b	8,380.33 ± 2,812.41b
GTR11	1,378.22 ± 89.94a	67.87 ± 8.73ab	213.23 ± 27.43ab	0.49 ± 0.04ab	2.57 ± 0.45b	3,208.67 ± 256.60a	5,920.00 ± 1,219.67ab	1,887.00 ± 289.77a	11,704.00 ± 1,970.74ab
GTS 16	1,045.40 ± 218.53b	55.53 ± 20.89ab	174.46 ± 65.64ab	0.52 ± 0.09ab	3.70a ± 0.23	2,159.00 ± 322.29bc	6,221.00 ± 1,041.28ab	1,827.67 ± 243.53a	11,798.00 ± 1,406.18ab

*Means within columns with different letters are significantly different as determined by Duncan’s multiple range test (p ≤ 0.05).*

*^*a*^This does not indicate the length of the root system per se but the cumulative length of all the primary, secondary, and tertiary roots and rootlets present in a root system.*

### Effect of Salinity and *O. tenuiflorum* Endophytes on Plant Growth and Biomass Accumulation

**FIGURE 8:**
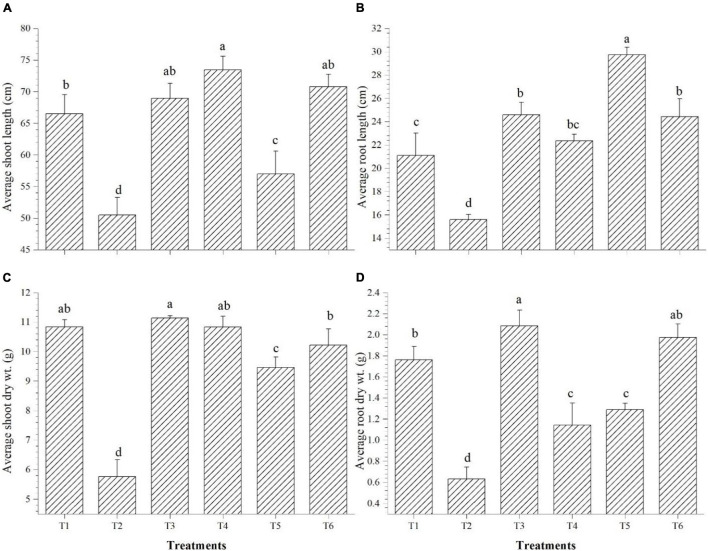
Effect of bacterial endophytes on the growth and biomass accumulation of tomato plants under elevated salinity (150 mM NaCl). **(A)** Average shoot length, **(B)** average root length, **(C)** average shoot dry weight, and **(D)** average root dry weight. T1, absolute control; T2, saline control; T3, BTL5; T4, GTR8; T5, GTR11; and T6, GTS16.

Shoot length was significantly improved over saline control in all the endophyte-inoculated plants; however, *Bacillus paralicheniformis* GTR11-inoculated plants have the lowest shoot length among endophyte-inoculated plants (Figure 8A). On the other hand, *Bacillus paralicheniformis* GTR11-inoculated plants had significantly higher root length than any other treatments (Figure 8B). Plants inoculated with *Bacillus safensis* BTL5 significantly influenced the root biomass after 45 days of transplanting compared to all other treatments (Figure 8D). The shoot biomass was also higher in the treatments inoculated with *Bacillus safensis* BTL5 but was at par with the absolute control treatment (T1) and treatments with *Bacillus haynesii* GTR8 inoculation (Figure 8C).

## Discussion

### Inter-Genera Colonization of *O. tenuiflorum* Endophytes in Tomato Roots

The results of the colonization study indicated that *O. tenuiflorum* endophytes could enter into tomato roots and colonize both outer and interior surfaces (Figure 1). Complex cross-talk is involved between plant and microorganism that affects endophytic colonization (Lahrmann et al., 2013); thus, exploring the potential endophytes having inter-genera colonization could widen the application of such potential cultures. The bacterial colonization in Figure 1 is indicated with white arrows. Strong colonization competence could have contributed to the successful colonization of *O. tenuiflorum* endophytes in tomato as earlier reported in the case of rice (Sahu et al., 2020a). There are reports indicating that inter-genera colonization contributes to plant fitness (Elbeltagy et al., 2001; Melnick et al., 2008; Abdallah et al., 2020). Endophytes having colonization in a wider range of hosts could be of greater significance for sustaining agriculture productivity.

### Modulation of Gene Expression

Modulation in the expression of *LKT*1, *NHX*1, *SOS*1, *LePIP*2, *SlERF*16, and *SlWRKY*39 genes was observed in tomato plants (Table 2), complementing the stress mitigation. This might be due to the induction of systemic tolerance by the applied endophytes that could have triggered the plants to overproduce the genes involved in salt stress alleviation (Vaishnav et al., 2020). Out of multiple genes involved in stress signaling, three structural genes, *LKT*1, *NHX*1, and *SOS*1, were chosen for this study since they code for K^+^ inward channel, Na^+^/K^+^ antiporter in vacuolar membrane, and Na^+^/K^+^ antiporter in roots, respectively (Hartje et al., 2000; Barragán et al., 2012; Wei et al., 2017). The fourth gene, *LePIP*2, encodes for plasma membrane aquaporins to maintain water homeostasis under salt stress. These four genes could have enhanced potassium influx and water balance and impart salt tolerance in tomato plants as validated in previous literatures (Qin et al., 2016; Wei et al., 2017; Molina-Montenegro et al., 2020). Thus, exploring these genes could provide insights for the induction of systemic tolerance by *O. tenuiflorum* endophytes. In plants, different *WRKY* and *ERF* transcription factors are extensively reported to be involved in drought and salt stress alleviation (Eulgem and Somssich, 2007; Gharsallah et al., 2016; Hong et al., 2018). The upregulation of *SlWRKY*39 and *SlERF*16 transcription factors in tomato plants could have activated multiple genes involved in ion homeostasis, oxidative stress pathway, photosynthesis, and enzyme activities as reported in different studies pertaining to salt stress (Sharma et al., 2010; Huang et al., 2012; Sun et al., 2015; Gharsallah et al., 2016; Hong et al., 2018). Our results showed that *Bacillus haynesii* GTR8, *Bacillus paralicheniformis* GTR11, and *Bacillus altitudinis* GTS16 inoculation enhanced the expression of *SlERF*16 and *SlWRKY*39 genes, correlating to the improvement in plant growth and development under elevated salinity. Similar to our results, improvement in salt stress tolerance was also reported in tomato plants over-expressing *ERF*16 and *SlWRKY*39 transcription factors (Gharsallah et al., 2016).

The expression of genes by endophyte inoculation was variable among the treatments, indicating the different modes of action of the applied inoculants. GTS 16 inoculation upregulated all the genes under study, whereas BTL5 could upregulate only *NHX*1. This might be due to difference in the modes of action among the applied endophytes. A similar complementation among the different microbial inoculants for improving plant performance was reported earlier (Shang and Liu, 2021). This could be a useful trait for the application of endophytic inoculants in a consortium.

### Cellular Osmotic Balance and Membrane Integrity Under Elevated Salinity

In the present study, oxidative stress protection could be correlated with enhanced accumulation of proline in the plants inoculated with endophytes *Bacillus paralicheniformis* GTR11 and *Bacillus altitudinis* GTS16 (Figure 3). Proline plays a dual role of antioxidant as well as osmoregulator and is significant in maintaining water homeostasis under stress. In the present study, both proline-induced osmoregulation and maintenance of root structure might play roles in maintaining the water status of salt-affected plants (Hayat et al., 2012; Qurashi and Sabri, 2013; Shafi et al., 2019). Decreased content of proline (as observed in T3 and T4) was also reported as a beneficial trait, indicating lowered salt stress in the plant system (Bhise et al., 2017). Similar effects were observed in total phenolics content (Figure 3A). Increased total phenolics content is reported as a salt stress-alleviating trait (Bhise et al., 2017). Golkar and Taghizadeh (2018) reported that phenolics are the main contributors of antioxidant activity in safflower at the cellular level apart from having a positive correlation with other compatible solutes contributing to the osmotic balance under elevated salinity. Plants of *Bacillus altitudinis* GTS16-inoculated treatment were found to have the lowest electrolyte leakage similar to the absolute control treatment, which indicates ion homeostasis and compatible solute production. Higher membrane integrity could be a result of the accumulation of osmoprotectants, production of enzymatic and non-enzymatic antioxidants, and reduced ROS generation. In similar lines, Amanifar et al. (2019) reported a decrease in electrolyte leakage due to the application of arbuscular mycorrhizal fungi under elevated salinity. There are other reports of improving membrane integrity under salt stress by inoculating stress-alleviating rhizobacteria (Sapre et al., 2021). Modulation of proline content and other compatible solutes due to the application of endophytes could also be one of the reasons for the reduction of ROS and increased membrane integrity.

### Effects of *O. tenuiflorum* Endophyte Inoculation on Different Reactive Oxygen Species Scavengers and Programmed Cell Death

Plants harbor antioxidant enzyme systems which protect them from the deleterious effect of very high levels of reactive oxygen species (ROS) generated due to stress (Kasote et al., 2015). The results from the enzymatic antioxidant activity indicated that the mechanisms for salt stress alleviation varied among different *O. tenuiflorum* endophytes (Figure 4). The enhancement in enzyme activities could be due to the triggering of the systemic mechanisms of the plant by endophytes (Sahu et al., 2019) by microbe-associated molecular patterns and other endophytic activities contributing to the induced systemic tolerance. There are various reports of endophytes improving the enzymatic antioxidant activity of plants by induced systemic tolerance (Halo et al., 2015; Vaishnav et al., 2020).

Triggering of PO, CAT, APx, and SOD activities by the induction of systemic tolerance could be a reason for the reduced sub-cellular accumulation of H_2_O_2_ and superoxide radicals in tomato leaves (Figure 5). Leaf staining revealed that the antioxidant system was triggered due to endophyte application, which could have contributed to the mitigation of salt stress. The antioxidant defense system of the plant was supplemented by endophyte inoculation, as reported in other studies in tomato (Halo et al., 2015; Sahu et al., 2019), under elevated salinity. Additionally, a lower sign of PCD was observed from *B. haynesii* GTR8-inoculated plants which could have contributed to the improved plant performance (Joseph and Jini, 2010). Endophytes supplementing the antioxidant machinery of a plant would be of greater economic significance for the alleviation of the secondary ill effects of stress.

### Na^+^ Transport and Accumulation in Plant Tissues

Reduction in Na^+^ transport and accumulation was found by the inoculation of *O. tenuiflorum* endophytes, especially in *Bacillus haynesii* GTR8, *Bacillus paralicheniformis* GTR11, and *Bacillus altitudinis* GTS16-inoculated treatments (Figure 6). Similar reports with the use of sodium Green^TM^ Tetra (tetramethylammonium) salt have shown a relationship with Na^+^ content of the plant tissue with differential exposures to NaCl (Wegner et al., 2011). This Sodium Green^TM^ Tetra (tetramethylammonium) salt is a visible light-excitable Na^+^ indicator and provides a brilliant spatial and temporal resolution of Na^+^ concentrations with sufficient sodium ions than other monovalent cations (Minta and Tsien, 1989). The spectral characteristics give the advantage of low cellular autofluorescence (Zhang and Melvin, 1996). The reduction in Na^+^ concentration of endophyte-inoculated plants could be due to the reduced sodium uptake, higher biomass accumulation, and ion compartmentalization by maintaining the Na^+^/K^+^ balance in cells. It also reflects the effects of the endophytes on the upregulation of structural (*LKT*1, *NHX*1, *SOS*1, and *LePIP*2) and regulatory genes (*SlERF*16 and *SlWRKY*39) which, in turn, reduce Na^+^ toxicity in the cells.

### *O. tenuiflorum* Endophytes Modulated Tomato Root Architecture Under Elevated Salinity

The plant roots are greatly affected by soil salinity (Maggio et al., 2005; Zhao et al., 2011). After exposure to salinity, the number of lateral roots decreases significantly (Julkowska et al., 2014). The present study also supports these findings where the root system architecture of tomato plants was significantly affected by salinity (150 mM NaCl). It is well documented that improvement in root parameters benefits plants in surviving under salt toxicity (Koevoets et al., 2016; Arif et al., 2019). In the present study, inoculation of *O. tenuiflorum* endophytes improved the root architecture (Table 3). Higher root projection area and surface area with a higher number of lateral roots in endophyte-inoculated plants might have helped the plants to mine water and nutrients more efficiently under osmotic stress caused by salinity.

Similar to our results, improvement in the root architecture by endophyte inoculation was also reported by other workers (Maggini et al., 2019; Muthukumar and Sulaiman, 2021; Verma et al., 2021b), specifically, improvement in root surface area (Irizarry and White, 2017; Yakti et al., 2018), root branching (Crush et al., 2004; Irizarry and White, 2017), root diameter (Martinuz et al., 2015), number of tips (Irizarry and White, 2017), etc. The improvement could be due to the production of a number of phytohormones, including IAA by the endophytes, that can modulate the endogenous levels of phytohormones in plants (Verma et al., 2021a). Increased levels of IAA due to the presence of endophytes can increase the root surface area, lateral root, and root hair formation (Vacheron et al., 2013; Zamioudis et al., 2015). Wang et al. (2015) reported that inoculation of endophytic *Bacillus* sp. LZR216 could influence auxin biosynthesis and transport, resulting in increased lateral root growth and reduced primary root elongation. The endophytes evaluated in this study could produce IAA (2.51–4.52 μg ml^–1^), which might have contributed toward the improvement of the root system architecture of tomato plants. However, the improvement in root growth can be independent of jasmonate, auxin, or ethylene signaling as well—for instance, the increased lateral root growth of *Arabidopsis* due to inoculation of *Bacillus siamensis* YC7012 has been attributed toward the production of volatile compounds (Hossain et al., 2019). Considering the previous findings and the results from the present study, it can be concluded that suitable endophyte inoculation can promote lateral root development and higher root length in tomato plants under high salt stress.

### Effect of Salinity and *O. tenuiflorum* Endophytes on Plant Growth and Biomass Accumulation

The increase in biomass accumulation by endophyte application (Figure 8) indicated the reduced effect of stress in the plants, due to which the plants could grow well and accumulate higher dry matter similar to the reports of Egamberdieva et al. (2016) and Vaishnav et al. (2020). The growth of the plants is basically dependent on the photosynthetic rates and availability of water and nutrients in the soil. The improvement in root architecture, reduction in ROS accumulation, and upregulation of the genes responsible for membrane transporters and other regulatory pathways by the *O. tenuiflorum* endophytes could have provided osmotic balance in plant cells and helped maintain normal growth under elevated salinity. High salinity negatively affects chlorophyll content and thus the total photosynthesis (Akcin and Yalcin, 2016; Chauhan et al., 2018). Therefore, enhancement in chlorophyll content could also have contributed to total biomass accumulation. Like rhizospheric microorganisms, endophytes could have also played a significant role in nutrient mobilization and uptake which, in turn, can improve the overall growth of plants. Secretion of organic acids, siderophores, by the endophytes helps in the sequestration of nutrients like phosphorus, potassium, zinc, etc. (Verma et al., 2021a). The improved growth and significant enhancement of biomass of pearl millet by *Bacillus cereus* EPP5 was due to the solubilization of nutrients, production of siderophores, and synthesis of IAA (Kushwaha et al., 2020). Similarly, the inoculation of P-solubilizing, diazotrophic *Bacillus methylotrophicus* CKAM to apple resulted in a significant increase in root and shoot growth (Mehta et al., 2014). Ismail et al. (2021) reported that inoculation of IAA-producing and P-solubilizing *Bacillus thuringiensis* PB2 and *Brevibacillus agri* PB5 resulted in increased biomass and yield traits in *Phaseolus vulgaris.* Therefore, the plant growth-promoting traits exhibited by the endophytes used in the present study might have additionally enhanced the performance of the plants under salt stress and improved the biomass accumulation.

### Complementary Roles of *O. tenuiflorum* Endophytes

The different endophytes used in this study have shown variable effects on plant growth. Here the effects of applied microbial inoculants on salt stress tolerance could be operated at least in two different modes: (i) endophyte could reduce the effect of salt toxicity (i.e., plant senses less salt stress) by effectively excluding and compartmentalizing the Na^+^ ions (Barragán et al., 2012; Wei et al., 2017), and (ii) once the plant senses salt toxicity, it activates the pathways for protecting plants by the formation of compatible solutes, ROS scavengers, and other compounds which can reduce the ill effects (Qurashi and Sabri, 2013; Halo et al., 2015). The salt stress-alleviating microbes could follow any of the abovementioned paths to complement the mechanism of salt tolerance of plants. Additionally, in mode (ii), since microbes have a vast metabolic diversity, they may supplement different mechanisms of plants as seen in T4; it has significantly enhanced TPC (Figure 3A) but has no effect on proline content among the saline treatments. Inoculation of T3 (*Bacillus safensis* BTL5 +150 mM NaCl) did not improve TPC and proline but has the lowest electrolyte leakage (Figure 3C), highest chlorophyll content (Figure 7), and highest root and shoot dry weight (Figures 8C,D). It has significantly improved different aspects of root architecture and also has a relatively higher expression of *NHX*1. It indicates the possibility of mix operating of modes (i) and (ii) in *Bacillus safensis* BTL5. On the other hand, inoculation of *Bacillus haynesii* GTR8 did not have much effect on root architecture but had the highest overexpression of *LePIP*2, *SOS*1, *SlERF*16, and *SlWRKY*39 genes, contributing to ionic balance, water homeostasis, and stress signaling. Additionally, *Bacillus paralicheniformis* GTR11 inoculation had a significantly higher effect on ascorbate peroxidase activity and thus reduced the accumulation of H_2_O_2_, and superoxide was observed. BTL5 and GTS16 inoculation had the lowest electrolyte leakage, whereas inoculation with GTR8 and GTS16 had the highest total phenolics and proline content, respectively. It is very crucial that analyzing the results revealed that the endophytes were complementary to each other. All four endophytes found complementing plant machinery for stress alleviation through different mechanisms. The results indicate that these endophytes could be suitable for application as consortium so as to complement each other in improving plant performance under salt stress.

## Conclusion

The results obtained from the present study suggested the protective roles of bacterial endophytes in tomato plants under elevated salinity. These endophytes could modulate the antioxidant machinery, root architecture, and ionic balance of the cells of plants to strengthen them against salt stress. Thus, the application of bacterial endophytes by harnessing the complementary roles of *Bacillus safensis* BTL5, *Bacillus haynesii* GTR8, *Bacillus paralicheniformis* GTR11, and *Bacillus altitudinis* GTS16 could be of greater significance in promoting plant growth and productivity in saline areas. The added features of the inter-genera colonization of endophytic isolates could add to the applicability and popularity of the strain by not limiting the choice of farmers to apply the inoculants. However, the field efficacy of these inoculants is yet to be tested. Exploring more endophytes having a wider host range could add to the pace of commercial endophytic inoculant development. Further dissecting the effects of endophytes on protein level could give a wider understanding of the mechanisms of the endophytes involved.

## Data Availability Statement

The original contributions presented in the study are included in the article/Supplementary Material, further inquiries can be directed to the corresponding author/s.

## Author Contributions

PSa conceived the idea and designed the experiments. PSa, SS, HC, BT, and RB performed the experiments. PSa, BS, and US analyzed the data. PSa, PSh, BS, HS, and AS wrote and edited the manuscript. AB has analyzed the gene expression data. All authors have reviewed the manuscript and have given approval to the final version.

## Conflict of Interest

The authors declare that the research was conducted in the absence of any commercial or financial relationships that could be construed as a potential conflict of interest.

## Publisher’s Note

All claims expressed in this article are solely those of the authors and do not necessarily represent those of their affiliated organizations, or those of the publisher, the editors and the reviewers. Any product that may be evaluated in this article, or claim that may be made by its manufacturer, is not guaranteed or endorsed by the publisher.

## References

[B1] Abdallah D. B. Krier F. Jacques P. Tounsi S. Frikha-Gargouri O. (2020). *Agrobacterium tumefaciens* C58 presence affects *Bacillus velezensis* 32a ecological fitness in the tomato rhizosphere. *Environ. Sci. Pollut. Res.* 27 28429–28437. 10.1007/s11356-020-09124-1 32415456

[B2] Abdelshafy Mohamad O. A. Ma J. B. Liu Y. H. Zhang D. Hua S. Bhute S. (2020). Beneficial endophytic bacterial populations associated with medicinal plant *Thymus vulgaris* alleviate salt stress and confer resistance to *Fusarium oxysporum*. *Front. Plant Sci.* 11:47. 10.3389/fpls.2020.00047 32117385 PMC7033553

[B3] Akcin A. Yalcin E. (2016). Effect of salinity stress on chlorophyll, carotenoid content, and proline in *Salicornia prostrata* Pall. and *Suaeda prostrata* Pall. subsp. prostrata (Amaranthaceae). *Rev. Bras. Bot.* 39 101–106. 10.1007/s40415-015-0218-y

[B4] Albdaiwi R. N. Khaymi-Horani H. Ayad J. Y. Alananbeh K. M. Kholoud M. Al-Sayaydeh R. (2019). Isolation and characterization of halotolerant plant growth-promoting rhizobacteria from durum wheat (*Triticum turgidum* subsp. durum) cultivated in saline areas of the dead sea region. *Front. Microbiol.* 10:1639. 10.3389/fmicb.2019.01639 31396175 PMC6664018

[B5] Amanifar S. Khodabandeloo M. Fard E. M. Askari M. S. Ashrafi M. (2019). Alleviation of salt stress and changes in glycyrrhizin accumulation by arbuscular mycorrhiza in liquorice (*Glycyrrhiza glabra*) grown under salinity stress. *Environ. Exp. Bot.* 160 25–34. 10.1016/j.envexpbot.2019.01.001

[B6] Arif M. R. Islam M. T. Robin A. H. K. (2019). Salinity stress alters root morphology and root hair traits in *Brassica napus*. *Plants* 8:192. 10.3390/plants8070192 31252515 PMC6681291

[B7] Arif Y. Singh P. Siddiqui H. Bajguz A. Hayat S. (2020). Salinity induced physiological and biochemical changes in plants: an omic approach towards salt stress tolerance. *Plant Physiol. Biochem.* 156 64–77. 10.1016/j.plaphy.2020.08.042 32906023

[B8] Ashraf M. Hasnain S. Berge O. Mahmood T. (2004). Inoculating wheat seedlings with exopolysaccharide-producing bacteria restricts sodium uptake and stimulates plant growth under salt stress. *Biol. Fertil. Soils* 40 157–162.

[B9] Baker C. J. Mock N. M. (1994). An improved method for monitoring cell death in cell suspension and leaf disc assays using Evans blue. *Plant Cell Tissue Organ Cult.* 39 7–12. 10.1007/bf00037585

[B10] Bano A. Fatima M. (2009). Salt tolerance in Zea mays (L) following inoculation with *Rhizobium* and *Pseudomonas*. *Biol. Fertil. Soils* 45 405–413. 10.1007/s00374-008-0344-9

[B11] Barragán V. Leidi E. O. Andrés Z. Rubio L. De Luca A. Fernández J. A. (2012). Ion exchangers NHX1 and NHX2 mediate active potassium uptake into vacuoles to regulate cell turgor and stomatal function in *Arabidopsis*. *Plant Cell* 24 1127–1142. 10.1105/tpc.111.095273 22438021 PMC3336136

[B12] Bhise K. K. Bhagwat P. K. Dandge P. B. (2017). Synergistic effect of *Chryseobacterium gleum* sp. SUK with ACC deaminase activity in alleviation of salt stress and plant growth promotion in Triticum aestivum L. *3 Biotech.* 7 1–13. 10.1007/s13205-017-0739-0 PMC5449284 28560646

[B13] Chauhan A. Rajput N. Kumar A. Verma J. S. Chaudhry A. K. (2018). Interactive effects of gibberellic acid and salt stress on growth parameters and chlorophyll content in oat cultivars. *J. Environmen. Biol.* 39 639–646. 10.22438/jeb/39/5/mrn-615

[B14] Chauhan P. S. Lata C. Tiwari S. Chauhan A. S. Mishra S. K. Agrawal L. (2019). Transcriptional alterations reveal *Bacillus amyloliquefaciens*-rice cooperation under salt stress. *Sci. Rep.* 9 1–13. 10.1016/j.plaphy.2013.01.020 31417134 PMC6695486

[B15] Chowdhary K. Kaushik N. (2015). Fungal endophyte diversity and bioactivity in the Indian medicinal plant *Ocimum sanctum* Linn. *PLoS One* 10:0141444. 10.1371/journal.pone.0141444 PMC4631451 26529087

[B16] Crush J. R. Popay A. J. Waller J. (2004). Effect of different Neotyphodium endophytes on root distribution of a perennial ryegrass (*Lolium perenne* L.) cultivar. *N. Z. J. Agric. Res.* 47 345–349.

[B17] Czarnocka W. Karpiński S. (2018). Friend or foe? Reactive oxygen species production, scavenging and signaling in plant response to environmental stresses. *Free Radic. Biol. Med.* 122 4–20. 10.1016/j.freeradbiomed.2018.01.011 29331649

[B18] Dodd I. C. Pérez-Alfocea F. (2012). Microbial amelioration of crop salinity stress. *J. Exp. Bot.* 636 3415–3428. 10.1093/jxb/ers033 22403432

[B19] Egamberdieva D. Jabborova D. Berg G. (2016). Synergistic interactions between Bradyrhizobium japonicum and the endophyte *Stenotrophomonas rhizophila* and their effects on growth, and nodulation of soybean under salt stress. *Plant Soil* 405 35–45. 10.1007/s11104-015-2661-8

[B20] Elbeltagy A. Nishioka K. Sato T. Suzuki H. Ye B. Hamada T. (2001). Endophytic colonization and in planta nitrogen fixation by a *Herbaspirillum* sp. isolated from wild rice species. *Appl. Environ. Microbiol.* 67 5285–5293. 10.1128/aem.67.11.5285-5293.2001 11679357 PMC93302

[B21] Eulgem T. Somssich I. E. (2007). Networks of WRKY transcription factors in defense signaling. *Curr. Opin. Plant Biol.* 10 366–371. 10.1016/j.pbi.2007.04.020 17644023

[B22] Fazal A. Bano A. (2016). Role of plant growth-promoting rhizobacteria (PGPR), biochar, and chemical fertilizer under salinity stress. *Commun. Soil Sci. Plant Anal.* 47 1985–1993. 10.1080/00103624.2016.1216562

[B23] Fujita M. Fujita Y. Noutoshi Y. Takahashi F. Narusaka Y. Yamaguchi-Shinozaki K. (2006). Crosstalk between abiotic and biotic stress responses: a current view from the points of convergence in the stress signaling networks. *Curr. Opin. Plant Biol.* 9 436–442. 10.1016/j.pbi.2006.05.014 16759898

[B24] Fukami J. de la Osa C. Ollero F. J. Megías M. Hungria M. (2018). Co-inoculation of maize with *Azospirillum brasilense* and *Rhizobium tropici* as a strategy to mitigate salinity stress. *Funct. Plant Biol.* 45 328–339. 10.1071/fp17167 32290956

[B25] Gharsallah C. Fakhfakh H. Grubb D. Gorsane F. (2016). Effect of salt stress on ion concentration, proline content, antioxidant enzyme activities and gene expression in tomato cultivars. *AoB Plants* 8:lw055. 10.1093/aobpla/plw055 PMC5091694 27543452

[B26] Golkar P. Taghizadeh M. (2018). In vitro evaluation of phenolic and osmolite compounds, ionic content, and antioxidant activity in safflower (*Carthamus tinctorius* L.) under salinity stress. *Plant Cell Tissue Organ Cult.* 134 357–368. 10.1007/s11240-018-1427-4

[B27] Halo B. A. Khan A. L. Waqas M. Al-Harrasi A. Hussain J. Ali L. (2015). Endophytic bacteria (*Sphingomonas* sp. LK11) and gibberellin can improve *Solanum lycopersicum* growth and oxidative stress under salinity. *J. Plant Interact.* 10 117–125. 10.1080/17429145.2015.1033659

[B28] Hartje S. Zimmermann S. Klonus D. Mueller-Roeber B. (2000). Functional characterisation of LKT1, a K+ uptake channel from tomato root hairs, and comparison with the closely related potato inwardly rectifying K+ channel SKT1 after expression in *Xenopus oocytes*. *Planta* 210 723–731. 10.1007/s004250050673 10805443

[B29] Hassani A. Azapagic A. Shokri N. (2020). Predicting long-term dynamics of soil salinity and sodicity on a global scale. *Proc. Natl. Acad. Sci. U.S.A.* 117 33017–33027. 10.1073/pnas.2013771117 33318212 PMC7776813

[B30] Hayat S. Hayat Q. Alyemeni M. N. Wani A. S. Pichtel J. Ahmad A. (2012). Role of proline under changing environments: a review. *Plant Signal. Behav.* 7 1456–1466. 10.4161/psb.21949 22951402 PMC3548871

[B31] Hong Y. Cui J. Liu Z. Luan Y. (2018). SpWRKY6 acts as a positive regulator during tomato resistance to *Phytophthora infestans* infection. *Biochem. Biophys. Res. Commun.* 506, 787–792. 30389138 10.1016/j.bbrc.2018.10.155

[B32] Hossain M. T. Khan A. Harun-Or-Rashid M. Chung Y. R. (2019). A volatile producing endophytic *Bacillus siamensis* YC7012 promotes root development independent on auxin or ethylene/jasmonic acid pathway. *Plant Soil* 439 309–324. 10.1007/s11104-019-04015-y

[B33] Huang S. Gao Y. Liu J. Peng X. Niu X. Fei Z. (2012). Genome-wide analysis of WRKY transcription factors in Solanum lycopersicum. *Mol. Genet. Genom.* 287, 495–513. 10.1007/s00438-012-0696-6 22570076

[B34] Irizarry I. White J. F. (2017). Application of bacteria from non-cultivated plants to promote growth, alter root architecture and alleviate salt stress of cotton. *J. Appl. Microbiol.* 122 1110–1120. 10.1111/jam.13414 28176460

[B35] Ismail M. A. Amin M. A. Eid A. M. Hassan S. E. D. Mahgoub H. A. Lashin I. (2021). Comparative study between exogenously applied plant growth hormones versus metabolites of microbial endophytes as plant growth-promoting for *Phaseolus vulgaris* L. *Cells* 10:1059. 10.3390/cells10051059 33946942 PMC8146795

[B36] Joseph B. Jini D. (2010). Salinity induced programmed cell death in plants: challenges and opportunities for salt-tolerant plants. *J. Plant Sci.* 5 376–390. 10.3923/jps.2010.376.390

[B37] Julkowska M. M. Hoefsloot H. C. Mol S. Feron R. de Boer G. J. Haring M. A. (2014). Capturing *Arabidopsis* root architecture dynamics with ROOT-FIT reveals diversity in responses to salinity. *Plant Physiol.* 166 1387–1402. 10.1104/pp.114.248963 25271266 PMC4226346

[B38] Kasote D. M. Katyare S. S. Hegde M. V. Bae H. (2015). Significance of antioxidant potential of plants and its relevance to therapeutic applications. *Int. J. Biol. Sci.* 11 982–991. 10.7150/ijbs.12096 26157352 PMC4495415

[B39] Khan M. A. Asaf S. Khan A. L. Adhikari A. Jan R. Ali S. (2020). Plant growth−promoting endophytic bacteria augment growth and salinity tolerance in rice plants. *Plant Biol.* 22 850–862. 10.1111/plb.13124 32329163

[B40] Khare N. Goyary D. Singh N. K. Shah P. Rathore M. Anandhan S. (2010). Transgenic tomato cv. Pusa Uphar expressing a bacterial mannitol-1-phosphate dehydrogenase gene confers abiotic stress tolerance. *Plant Cell Tissue Organ Cult.* 103 267–277. 10.1007/s11240-010-9776-7

[B41] Koevoets I. T. Venema J. H. Elzenga J. T. Testerink C. (2016). Roots withstanding their environment: exploiting root system architecture responses to abiotic stress to improve crop tolerance. *Front. plant sci.* 7:1335. 10.3389/fpls.2016.01335 27630659 PMC5005332

[B42] Kumar K. Gambhir G. Dass A. Tripathi A. K. Singh A. Jha A. K. (2020). Genetically modified crops: current status and future prospects. *Planta* 251 1–27. 10.1007/s00425-020-03372-8 32236850

[B43] Kushwaha P. Kashyap P. L. Bhardwaj A. K. Kuppusamy P. Srivastava A. K. Tiwari R. K. (2020). Bacterial endophyte mediated plant tolerance to salinity: growth responses and mechanisms of action. *World J. Microbiol. Biotechnol.* 36 1–16. 10.1007/978-3-319-90318-7_1 31997078

[B44] Lahrmann U. Ding Y. Banhara A. Rath M. Hajirezaei M. R. Döhlemann S. (2013). Host-related metabolic cues affect colonization strategies of a root endophyte. *Proc. Natl. Acad. Sci. U.S.A.* 110 13965–13970. 10.1073/pnas.1301653110 23918389 PMC3752250

[B45] Lee G. W. Lee K. J. Chae J. C. (2016). *Herbaspirillum* sp. strain GW103 alleviates salt stress in *Brassica rapa* L. ssp. pekinensis. *Protoplasma* 253 655–661. 10.1007/s00709-015-0872-8 26358119

[B46] Lee Y. Krishnamoorthy R. Selvakumar G. Kim K. Sa T. (2015). Alleviation of salt stress in maize plant by co-inoculation of arbuscular mycorrhizal fungi and *Methylobacterium oryzae* CBMB20. *J. Korean Soc. Appl. Bi.* 58 533–540. 10.1007/s13765-015-0072-4

[B47] Livak K. J. Schmittgen T. D. (2001). Analysis of relative gene expression data using real-time quantitative PCR and the 2(-Delta C(T)) method. *Methods* 25 402–408. 10.1006/meth.2001.1262 11846609

[B48] López-Gómez M. Hidalgo-Castellanos J. Marín-Peńa A. J. Herrera-Cervera J. A. (2019). “Relationship between polyamines and osmoprotectants in the response to salinity of the legume–rhizobia symbiosis,” in *Osmoprotectant-Mediated Abiotic Stress Tolerance in Plants*, eds Hossain M. Kumar V. Burritt D. Fujita M. Mäkelä P. (Cham: Springer), 269–285. 10.1007/978-3-030-27423-8_13

[B49] Maggini V. Mengoni A. Gallo E. R. Biffi S. Fani R. Firenzuoli F. (2019). Tissue specificity and differential effects on in vitro plant growth of single bacterial endophytes isolated from the roots, leaves and rhizospheric soil of *Echinacea purpurea*. *BMC Plant Biol.* 19:284. 10.1186/s12870-019-1890-z 31253081 PMC6598257

[B50] Maggio A. De Pascale S. Ruggiero C. Barbieri G. (2005). Physiological response of field-grown cabbage to salinity and drought stress. *Eur. J. Agron.* 23 57–67. 10.1016/j.eja.2004.09.004

[B51] Malick C. P. Singh M. B. (1980). *Plant Enzymology and Histo Enzymology.* New Delhi: Kalyani publishers.

[B52] Martinuz A. Zewdu G. Ludwig N. Grundler F. Sikora R. A. Schouten A. (2015). The application of *Arabidopsis thaliana* in studying tripartite interactions among plants, beneficial fungal endophytes and biotrophic plant-parasitic nematodes. *Planta* 241 1015–1025. 10.1007/s00425-014-2237-5 25548000

[B53] Mehta P. Walia A. Kakkar N. Shirkot C. K. (2014). Tricalcium phosphate solubilisation by new endophyte *Bacillus methylotrophicus* CKAM isolated from apple root endosphere and its plant growth-promoting activities. *Acta Physiol. Plant* 36 2033–2045. 10.1007/s11738-014-1581-1

[B54] Melnick R. L. Zidack N. K. Bailey B. A. Maximova S. N. Guiltinan M. Backman P. A. (2008). Bacterial endophytes: *Bacillus* spp. from annual crops as potential biological control agents of black pod rot of cacao. *Biol. control* 46 46–56. 10.1016/j.biocontrol.2008.01.022

[B55] Minta A. Tsien R. Y. (1989). Fluorescent indicators for cytosolic sodium. *J. Biol. Chem.* 264 19449–19457. 10.1016/s0021-9258(19)47321-3 2808435

[B56] Molina-Montenegro M. A. Acuña-Rodríguez I. S. Torres-Díaz C. Gundel P. E. Dreyer I. (2020). Antarctic root endophytes improve physiological performance and yield in crops under salt stress by enhanced energy production and Na+ sequestration. *Sci. Rep.* 10:5819. 10.1038/s41598-020-62544-4 PMC7118072 32242034

[B57] Muthukumar T. Sulaiman M. R. (2021). Root Endophytic *Nectria haematococca* influences growth, root architecture and phosphorus content of green gram in different substrates. *Proc. Natl. Acad. Sci. India Sect. B Biol. Sci.* 91 131–138. 10.1007/s40011-020-01195-x

[B58] Nadeem M. Ali M. Kubra G. Fareed A. Hasan H. Khursheed A. (2020). “Role of osmoprotectants in salinity tolerance in wheat,” in *Climate Change and Food Security with Emphasis on Wheat*, eds Ozturk M. Gul A. (Cambridge, MA: Academic Press), 93–106. 10.1016/b978-0-12-819527-7.00006-6

[B59] Pan T. Liu M. Kreslavski V. D. Zharmukhamedov S. K. Nie C. Yu M. (2020). Non-stomatal limitation of photosynthesis by soil salinity. *Crit. Rev. Environ. Sci. Technol.* 51 1–35.

[B60] Qadir M. Schubert S. Ghafoor A. Murtaza G. (2001). Amelioration strategies for sodic soils: a review. *Land Degrad. Dev.* 12 357–386. 10.1002/ldr.458

[B61] Qin Y. Druzhinina I. S. Pan X. Yuan Z. (2016). Microbially mediated plant salt tolerance and microbiome-based solutions for saline agriculture. *Biotechnol. Adv.* 34 1245–1259. 10.1016/j.biotechadv.2016.08.005 27587331

[B62] Qurashi A. W. Sabri A. N. (2013). Osmolyte accumulation in moderately halophilic bacteria improves salt tolerance of chickpea. *Pak. J. Bot.* 45 1011–1016.

[B63] Rao M. V. Davis K. R. (1999). Ozone-induced cell death occurs via two distinct mechanisms in Arabidopsis: the role of salicylic acid. *Plant J.* 17, 603–614. 10230060 10.1046/j.1365-313x.1999.00400.x

[B64] Sadasivam S. Manickam A. (1996). *Biochemical Methods for Agricultural Sciences.* New Delhi: New Age International (P) Ltd., 1–97.

[B65] Sahu P. K. Singh S. Gupta A. Singh U. B. Brahmaprakash G. P. Saxena A. K. (2019). Antagonistic potential of bacterial endophytes and induction of systemic resistance against collar rot pathogen *Sclerotium rolfsii* in tomato. *Biol. Control* 137:104014. 10.1016/j.biocontrol.2019.104014

[B66] Sahu P. K. Thomas P. Singh S. Gupta A. (2020b). Taxonomic and functional diversity of cultivable endophytes with respect to the fitness of cultivars against *Ralstonia solanacearum*. *J. Plant Dis. Protect.* 127 1–10. 10.1007/s41348-020-00320-2

[B67] Sahu P. K. Singh S. Gupta A. R. Gupta A. Singh U. B. Manzar N. (2020a). Endophytic bacilli from medicinal-aromatic perennial Holy basil (*Ocimum tenuiflorum* L.) modulate plant growth promotion and induced systemic resistance against *Rhizoctonia solani* in rice (*Oryza sativa* L.). *Biol. Control* 150:104353. 10.1016/j.biocontrol.2020.104353

[B68] Sakamoto H. Matsuda O. Iba K. (2008). ITN1, a novel gene encoding an ankyrin-repeat protein that affects the ABA-mediated production of reactive oxygen species and is involved in salt-stress tolerance in *Arabidopsis thaliana*. *Plant J.* 56 411–422. 10.1111/j.1365-313x.2008.03614.x 18643991

[B69] Sapre S. Gontia-Mishra I. Tiwari S. (2021). Plant growth-promoting rhizobacteria ameliorates salinity stress in pea (*Pisum sativum*). *J. Plant Growth Regul.* 1–10. 10.1007/s00344-021-10329-y33649694

[B70] Shafi A. Zahoor I. Mushtaq U. (2019). “Proline accumulation and oxidative stress: diverse roles and mechanism of tolerance and adaptation under salinity stress,” in *Salt Stress, Microbes, and Plant Interactions: Mechanisms and Molecular Approaches*, eds Akhtar Sayeed M. (Singapore: Springer), 269–300. 10.1007/978-981-13-8805-7_13

[B71] Shang J. Liu B. (2021). Application of a microbial consortium improves the growth of *Camellia sinensis* and influences the indigenous rhizosphere bacterial communities. *J. Appl. Microbiol.* 130 2029–2040. 10.1111/jam.14927 33170985

[B72] Sharma M. K. Kumar R. Solanke A. U. Sharma R. Tyagi A. K. Sharma A. K. (2010). Identification, phylogeny, and transcript profiling of ERF family genes during development and abiotic stress treatments in tomato. *Mol. Genet. Genom.* 284, 455–475. 10.1007/s00438-010-0580-1 20922546

[B73] Shekhawat S. Shah G. (2013). Isolation, characterization and determinaton of antibacterial activity of bacterial and fungal endophytes from *Ocimum sanctum* and phytochemical analysis. *Fungal Divers.* 33 87–100.

[B74] Shih M. C. Heinrich R. Goodman H. M. (1992). Cloning and chromosomal mapping of nuclear genes encoding chloroplast and cytosolic glyceraldehyde-3-phosphate-dehydrogenase from Arabidopsis thaliana. *Gene* 119 317–319. 10.1016/0378-1119(92)90290-6 1398114

[B75] Shiota H. Sudoh T. Tanaka I. (2006). Expression analysis of genes encoding plasma membrane aquaporins during seed and fruit development in tomato. *Plant Sci.* 171 277–285. 10.1016/j.plantsci.2006.03.021

[B76] Singh D. Chaudhuri P. K. (2018). A review on phytochemical and pharmacological properties of Holy basil (*Ocimum sanctum* L.). *Ind. Crops Prod.* 118 367–382. 10.1016/j.indcrop.2018.03.048

[B77] Singh R. P. Jha P. N. (2016). A halotolerant bacterium *Bacillus licheniformis* HSW-16 augments induced systemic tolerance to salt stress in wheat plant (*Triticum aestivum*). *Front. Plant Sci.* 7:1890. 10.3389/fpls.2016.01890 28018415 PMC5159429

[B78] Singh R. P. Jha P. N. (2017). Analysis of fatty acid composition of PGPR *Klebsiella* sp. SBP-8 and its role in ameliorating salt stress in wheat. *Symbiosis* 73 213–222. 10.1007/s13199-017-0477-4

[B79] Stiefel P. Schmidt-Emrich S. Maniura-Weber K. Ren Q. (2015). Critical aspects of using bacterial cell viability assays with the fluorophores SYTO9 and propidium iodide. *BMC Microbiol.* 15:36. 10.1186/s12866-015-0376-x 25881030 PMC4337318

[B80] Sun X. C. Gao Y. F. Li H. R. Yang S. Z. Liu Y. S. (2015). Over-expression of SlWRKY39 leads to enhanced resistance to multiple stress factors in tomato. *J. Plant Biol.* 58, 52–60.

[B81] Taufiq M. M. J. Darah I. (2018). Fungal endophytes isolated from the leaves of a medicinal plant, *Ocimum sanctum* Linn and evaluation of their antimicrobial activities. *Afr. J. Microbiol. Res.* 12 616–622. 10.5897/ajmr2018.8812

[B82] Uga Y. (2021). Challenges to design-oriented breeding of root system architecture adapted to climate change. *Breed. Sci.* 71 3–12. 10.1270/jsbbs.20118 33762871 PMC7973499

[B83] Vacheron J. Desbrosses G. Bouffaud M. L. Touraine B. MoënneLoccoz Y. Muller D. (2013). Plant growth-promoting rhizobacteria and root system functioning. *Front. Plant Sci* 4:356. 10.3389/fpls.2013.00356 24062756 PMC3775148

[B84] Vaishnav A. Singh J. Singh P. Rajput R. S. Singh H. B. Sarma B. K. (2020). *Sphingobacterium* sp. BHU-AV3 induces salt tolerance in tomato by enhancing antioxidant activities and energy metabolism. *Front. Microbiol.* 11:443. 10.3389/fmicb.2020.00443 32308647 PMC7145953

[B85] Verma S. K. Sahu P. K. Kumar K. Pal G. Gond S. K. Kharwar R. N. White J. F. (2021b). Endophyte roles in nutrient acquisition, root system architecture development and oxidative stress tolerance. *J. Appl. Microbiol.* 1–17. 10.1111/jam.15111 33893707

[B86] Verma S. Chakdar H. Kumar M. Varma A. Saxena A. K. (2021a). Microorganisms as a sustainable alternative to traditional biofortification of iron and zinc: status and prospect to combat hidden hunger. *J. Soil Sci. Plant Nutr.* 21 1700–1717. 10.1007/s42729-021-00473-5

[B87] Wang J. Zhang Y. Li Y. Wang X. Nan W. Hu Y. (2015). Endophytic microbes *Bacillus* sp. LZR216-regulated root development is dependent on polar auxin transport in *Arabidopsis* seedlings. *Plant Cell Rep.* 34 1075–1087. 10.1007/s00299-015-1766-0 25700982

[B88] Wegner L. H. Stefano G. Shabala L. Rossi M. Mancuso S. Shabala S. (2011). Sequential depolarization of root cortical and stelar cells induced by an acute salt shock–implications for Na+ and K+ transport into xylem vessels. *Plant Cell Environ.* 34 859–869. 10.1111/j.1365-3040.2011.02291.x 21332511

[B89] Wei D. Zhang W. Wang C. Meng Q. Li G. Chen T. H. (2017). Genetic engineering of the biosynthesis of glycinebetaine leads to alleviate salt-induced potassium efflux and enhances salt tolerance in tomato plants. *Plant Sci.* 257 74–83. 10.1016/j.plantsci.2017.01.012 28224920

[B90] Witham F. H. Blaydes D. F. Devlin R. M. (1971). *Experiments in Plant Physiology.* New York, NY: Van Nostrand, 245.

[B91] Yakti W. Kovacs G. M. Vagi P. Franken P. (2018). Impact of dark septate endophytes on tomato growth and nutrient uptake. *Plant Ecol. Divers.* 11 637–648. 10.1080/17550874.2019.1610912

[B92] Yasin N. A. Akram W. Khan W. U. Ahmad S. R. Ahmad A. Ali A. (2018). Halotolerant plant-growth promoting rhizobacteria modulate gene expression and osmolyte production to improve salinity tolerance and growth in *Capsicum annum* L. *Environ. Sci. Pollut. Res.* 25 23236–23250. 10.1007/s11356-018-2381-8 29869207

[B93] Zamioudis C. Korteland J. Van Pelt J. A. van Hamersveld M. Dombrowski N. Bai Y. (2015). Rhizobacterial volatiles and photosynthesis-related signals coordinate MYB 72 expression in *Arabidopsis* roots during onset of induced systemic resistance and iron deficiency responses. *Plant J.* 84 309–322. 10.1111/tpj.12995 26307542 PMC5019235

[B94] Zhang G. H. Melvin J. E. (1996). Na+-dependent release of Mg2+ from an intracellular pool in rat sublingual mucous acini. *J. Biol. Chem.* 271 29067–29072. 10.1074/jbc.271.46.29067 8910560

[B95] Zhang H. Kim M. S. Sun Y. Dowd S. E. Shi H. Paré P. W. (2008). Soil bacteria confer plant salt tolerance by tissue-specific regulation of the sodium transporter HKT1. *Mol. Plant Microbe Interact.* 21 737–744. 10.1094/mpmi-21-6-0737 18624638

[B96] Zhao Y. Wang T. Zhang W. Li X. (2011). SOS3 mediates lateral root development under low salt stress through regulation of auxin redistribution and maxima in *Arabidopsis*. *New Phytol.* 189 1122–1134. 10.1111/j.1469-8137.2010.03545.x 21087263

