# Supplementary material for: Inter-Genera Colonization of Ocimum tenuiflorum Endophytes in Tomato and Their Complementary Effects on Na+/K+ Balance, Oxidative Stress Regulation, and Root Architecture Under Elevated Soil Salinity
Source: Front Microbiol. 2021 Oct 18;12:744733. doi: 10.3389/fmicb.2021.744733 (PMC8558678; doi:10.3389/fmicb.2021.744733)
Supplement: Supplementary file 1 [file Table_1.DOCX]

**Supplementary data 1: Growth promoting attributes of *O. tenuiflorum* endophytes**

| Endophytes | P-solubilization (mg/L) | IAA Production (µg/ml) | Zn solubilization | Ammonia production |
| --- | --- | --- | --- | --- |
| BTL 5 | 15.33 | 4.52 | + | - |
| GTR8 | 22.54 | 2.51 | ++ | + |
| GTR11 | 24.96 | 3.36 | + | + |
| GTS 16 | 26.36 | 3.21 | ++ | + |
